# Probing the quality control mechanism of the *Escherichia coli* twin-arginine translocase with folding variants of a *de novo*–designed heme protein

**DOI:** 10.1074/jbc.RA117.000880

**Published:** 2018-03-20

**Authors:** George A. Sutherland, Katie J. Grayson, Nathan B. P. Adams, Daphne M. J. Mermans, Alexander S. Jones, Angus J. Robertson, Dirk B. Auman, Amanda A. Brindley, Fabio Sterpone, Pierre Tuffery, Philippe Derreumaux, P. Leslie Dutton, Colin Robinson, Andrew Hitchcock, C. Neil Hunter

**Affiliations:** From the ‡ Department of Molecular Biology and Biotechnology, University of Sheffield, Sheffield S10 2TN, United Kingdom,; the § School of Biosciences, University of Kent, Canterbury CT2 7NJ, United Kingdom,; the ¶ Department of Biochemistry and Biophysics, University of Pennsylvania, Philadelphia, Pennsylvania 19104,; the ‖ Laboratoire de Biochimie Théorique, UPR 9080 CNRS, Université Paris Diderot, Sorbonne Paris Cité, 75005 Paris, France, and; ** INSERM U973, Université Paris Diderot, Sorbonne Paris Cité, 75013 Paris, France

**Keywords:** protein design, protein folding, Escherichia coli (E. coli), protein translocation, biotechnology, maquette, protein export, protein quality control, Tat system, twin-arginine translocase

## Abstract

Protein transport across the cytoplasmic membrane of bacterial cells is mediated by either the general secretion (Sec) system or the twin-arginine translocase (Tat). The Tat machinery exports folded and cofactor-containing proteins from the cytoplasm to the periplasm by using the transmembrane proton motive force as a source of energy. The Tat apparatus apparently senses the folded state of its protein substrates, a quality-control mechanism that prevents premature export of nascent unfolded or misfolded polypeptides, but its mechanistic basis has not yet been determined. Here, we investigated the innate ability of the model *Escherichia coli* Tat system to recognize and translocate *de novo*–designed protein substrates with experimentally determined differences in the extent of folding. Water-soluble, four-helix bundle maquette proteins were engineered to bind two, one, or no heme *b* cofactors, resulting in a concomitant reduction in the extent of their folding, assessed with temperature-dependent CD spectroscopy and one-dimensional ^1^H NMR spectroscopy. Fusion of the archetypal N-terminal Tat signal peptide of the *E. coli* trimethylamine-*N*-oxide (TMAO) reductase (TorA) to the N terminus of the protein maquettes was sufficient for the Tat system to recognize them as substrates. The clear correlation between the level of Tat-dependent export and the degree of heme *b*–induced folding of the maquette protein suggested that the membrane-bound Tat machinery can sense the extent of folding and conformational flexibility of its substrates. We propose that these artificial proteins are ideal substrates for future investigations of the Tat system's quality-control mechanism.

## Introduction

The transport of proteins across membranes is one of the great challenges faced by the cell. In prokaryotes, two major pathways are used to achieve protein translocation across the cytoplasmic (inner) membrane. The general secretion (Sec)^9^ pathway transports proteins in an unfolded configuration using energy provided by both ATP hydrolysis and the transmembrane proton gradient (1). In contrast, the twin-arginine translocase (Tat) system transports fully folded proteins (2, 3) and is energized solely by the transmembrane proton gradient.

The majority of Tat substrates are cofactor-containing proteins that require assembly in the cytoplasm (4–6) including those that fold too quickly for Sec transport (7) and those that assemble into oligomeric complexes (8). Proteins translocated by the Tat pathway have an N-terminal signal sequence characterized by a twin-arginine (RR) motif (9); the signal sequence is cleaved from the precursor protein during or immediately after translocation, releasing the mature protein into the periplasm.

How components of the Tat machinery assess the folding state of a protein substrate remains poorly understood (6, 10, 11). A quality control or proofreading mechanism could exist to prevent futile export of misfolded or misassembled proteins (12–14). In *Escherichia coli* the Tat apparatus comprises TatA/B family proteins and the TatC protein. Single point mutations in either TatA or TatC were identified that allow translocation of unfolded substrates, and point mutations in both TatB and TatC enabled export of a broader range of unfolded substrates, suggesting that the TatABC subunits cooperatively assess the folding state of proteins independently of protein translocation (15).

Richter *et al.* (16) showed that small, unstructured hydrophilic FG repeat proteins could be exported by the Tat system, and that the presence of hydrophobic surface patches was sufficient to abort transport, raising the possibility that the Tat system screens proteins based on their surface hydrophobicity. It has been reported that the length of the unstructured FG repeat polypeptide dramatically affects Tat export, with longer regions abolishing Tat export altogether (17). Conversely, Jones *et al.* (18) recently reported that the Tat system was surprisingly tolerant of hydrophobic patches on the surface of structured single-chain variable fragment proteins, and export efficiency was increased with greater structural rigidity. Chaperones may also prevent export of a protein until cofactor insertion has taken place (19–21), and mutants incapable of cofactor binding are rapidly degraded once in contact with the Tat machinery (22).

To further investigate the Tat quality-control mechanism, we used maquettes, which are simple, repetitive protein structures designed *de novo* from first principles with minimal reference to natural protein structures (23–26). As such structures contain unnecessary complexity, accumulated from perpetual rounds of blind natural selection (27), altering protein residues can have unpredictable effects on protein structure and dynamics. In contrast, the role of each amino acid in the simple maquette structure has been rationalized at the outset, so changes to structure and function become more predictable.

**Figure 1.**
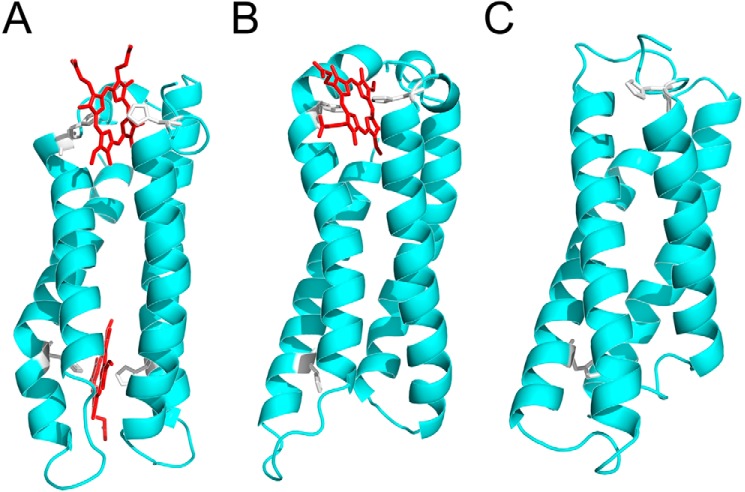
**Structural models of the BT6 maquette proteins used in this study.** *A,* BT6 (see Ref. 28) coordinates two heme *b* molecules (*red*) using four histidine ligands (*white*). *B,* in BT6M1 the H53A substitution means the protein can only coordinate one heme *b. C,* in BT6M0 the double H53A/H88A substitution prevents heme binding. All images were taken from 50-ns trajectories.

The maquettes used in this study are based on the BT6 maquette developed by Farid *et al.* (28), and consist of loops linking four largely identical α-helices enclosing a water-excluding cavity that can accommodate cofactors (Fig. 1*A–C*). Histidine residues within the maquette cavity ligate hemes, producing artificial proteins resembling *b*-type cytochromes and myoglobin (29, 30).

Here, three different maquette variants were utilized, each with a different heme *b* binding capacity (Fig. 1). Using nuclear magnetic resonance (NMR) and circular dichroism (CD) spectroscopy we show that binding two, one, or no hemes (Fig. 1, *A–C*, respectively) imparts changes in the extent of folding of the maquette variants. The archetypal trimethylamine-*N*-oxide reductase (TorA) Tat signal peptide was fused to the N terminus of the three proteins and the *E. coli* Tat machinery was challenged to differentiate between the folding variants. We show that the Tat apparatus is able to sense the conformational flexibility of the different maquette substrates, and that increasingly well-folded maquettes are exported with enhanced efficiency.

## Results

### Production of heme-reconstituted maquettes

The aim of this study was to test whether the *E. coli* Tat system could recognize and export a *de novo*–designed di-heme protein, and then to use variants of this protein with experimentally confirmed differences in conformational flexibility to test if the Tat apparatus selectively processes the more folded proteins.

As described above and depicted in the computationally-generated structures presented in Fig. 1, three variants of the BT6 maquette were produced, facilitating the incorporation of two (BT6), one (BT6M1), or no (BT6M0) heme *b* cofactors. The requirement for bis-histidine ligation of heme *b* in the artificial constructs enabled the generation of the one and no heme-binding variants through H53A, and H53A/H88A (histidine to alanine) point mutations, respectively (Table 1). The computational models in Fig. 1 extend the theoretical structure of the BT6 maquette outlined by Farid *et al.* (28). They are included to illustrate the apo- and heme-maquette designs used as Tat substrates, and are not intended as a replacement for atomically-accurate experimental structures.

**Table 1:** **Amino acid sequences of maquettes and signal peptides used in this study**

Name	Sequence*^a^*	Details
BT6	MGGDGENLYFQG	Di-heme binding
	EIWKQ**H**EDALQKFEEALNQFEDLKQLGGSGSGSGG	
	EIWKQ**H**EDALQKFEEALNQFEDLKQLGGSGSGSGG	
	EIWKQ**H**EDALQKFEEALNQFEDLKQLGGSGSGSGG	
	EIWKQ**H**EDALQKFEEALNQFEDLK	
BT6M1	MGGDGENLYFQG	Single heme binding due to removal of 1 coordinating histidine residues
	EIWKQ**H**EDALQKFEEALNQFEDLKQLGGSGSGSGG	
	EIWKQAEDALQKFEEALNQFEDLKQLGGSGSGSGG	
	EIWKQ**H**EDALQKFEEALNQFEDLKQLGGSGSGSGG	
	EIWKQ**H**EDALQKFEEALNQFEDLK	
BT6M0	MGGDGENLYFQG	No heme binding due to removal of 2 coordinating histidine residues
	EIWKQ**H**EDALQKFEEALNQFEDLKQLGGSGSGSGG	
	EIWKQAEDALQKFEEALNQFEDLKQLGGSGSGSGG	
	EIWKQAEDALQKFEEALNQFEDLKQLGGSGSGSGG	
	EIWKQ**H**EDALQKFEEALNQFEDLK	
TorA	MNNNDLFQAS***RR***RFLAQLGGLTVAGMLGPSLLTPRRATAAQAA	TorA signal peptide for periplasmic localization by the Tat system
TorA R12/13K	MNNNDLFQAS***KK***RFLAQLGGLTVAGMLGPSLLTPRRATAAQAA	R12K/R13K TorA signal peptide
PelB	MKYLLPTAAAGLLLLAAQPAMA	PelB signal peptide for periplasmic localization by Sec system

*^a^* Heme coordinating histidine residues are shown in bold. The twin-arginine motif of the Tat signal peptide is shown in bold italics.

**Figure 2.**
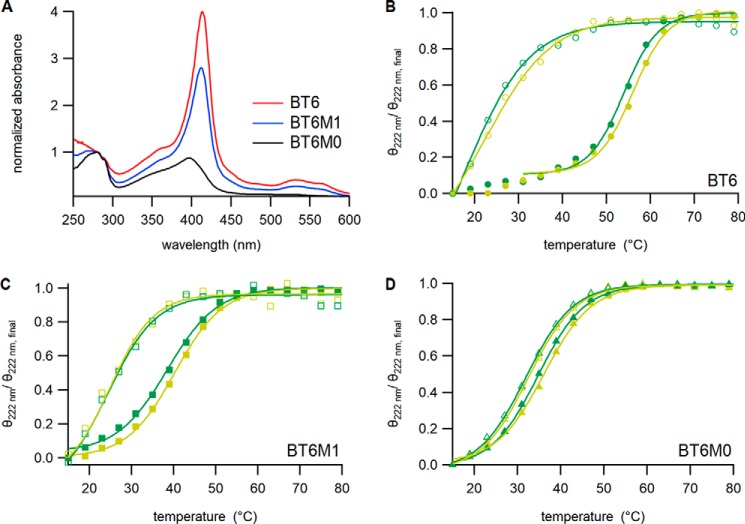
**Spectroscopic analysis of apo- and heme-reconstituted maquette proteins.** *A,* UV-visible absorption spectra of heme-reconstituted maquette samples normalized to absorbance at 280 nm. *B–D,* normalized melting (*yellow*) and refolding (*green*) of secondary structure measured as the reduction in CD ellipticity at 222 nm across a temperature gradient in the absence (*open markers*) or presence (*solid markers*) of heme. Ellipticity was recorded every 1 °C, but only every third data point is shown for clarity. *Lines* are theoretical and described by a Boltzmann distribution (see “Experimental procedures”). Melting (*T_m_*) and refolding (*T_f_*) temperatures are reported in Table 2.

Maquettes are typically overproduced in *E. coli* in large amounts, with bound heme largely absent following purification (see “Experimental procedures”) because the native tetrapyrrole biosynthesis pathway cannot keep pace with the induced synthesis of maquette (Fig. S1*A*). Apoproteins were reconstituted *in vitro* with an excess of heme and unbound pigment was removed by anion exchange chromatography. The Soret absorption bands for the BT6, BT6M1, and BT6M0 maquettes were normalized for protein concentration (absorbance at 280 nm) and had maxima at 413, 412, and 396 nm, respectively (Fig. 2*A*), with the amplitudes corresponding to binding two, one, and no hemes, respectively. The weak, blue-shifted absorption of the BT6M0 maquette suggested a low level of adventitiously bound heme. We compared heme absorption in the maquette-bound, and solvated (buffer or DMSO) states, which had respective maxima at 413, 404, and 384 nm (Fig. S1*B*). The blue-shifted absorption maxima for solvated heme are consistent with the weak, blue-shifted absorption for BT6M0 (Fig. 2*A*) arising from residual, weakly bound heme; ligation into the internal cavity of BT6 and BT6M1 causes a red-shift absorption of the heme.

### Thermostability and folding of maquette variants

Temperature-dependent CD spectroscopy was performed to assess the effect of heme ligation on the thermal stability of each maquette scaffold. At 15 °C the CD spectra of all three maquette variants, with or without bound heme, were typical of α-helical structures (Fig. S2, *black lines*). Likewise, irrespective of heme binding heating to 80 °C resulted in spectra typical of that of a denatured protein (31) (Fig. S2, from *black* to *red* to *gray lines*).

Ellipticity at 222 nm was monitored during temperature cycling from 15 to 80 to 15 °C to observe denaturing and subsequent re-folding of protein (Fig. 2, *B–D*). For BT6, heme incorporation displaced the midpoint of the melting curve from 22 to 56 °C, the 34 °C difference indicating increased stabilization following heme binding. The same procedure with BT6M1 increased stabilization by only 16 °C (25 to 41 °C), and had very little effect on the BT6M0 scaffold (32 to 35 °C) (Table 2). Thus, bis-histidine ligation of two hemes within the maquette cavity of BT6 significantly stabilizes the four-helix bundle structure, and there is a smaller effect with only one bis-histidine ligation in BT6M1. In the case of BT6M0, where heme ligation is not possible, there is correspondingly no significant stabilization of maquette structure in the presence of heme.

**Table 2:** **Melting temperatures (*T_m_*) and folding temperatures (*T_f_*) of maquettes in the absence (apo) or presence of heme**

Maquette	Apo		+Heme	
	*T_m_**^a^*	*T_f_*	*T_m_*	*T_f_*
	°*C*		°*C*	
BT6	22	17	56	54
BT6M1	25	24	41	39
BT6M0	32	32	35	36

*^a^* The *T_m_* and *T_f_* (temperature at which 50% of the protein is unfolded or folded, respectively) values were determined from Boltzman distribution fits as shown in Fig. 2, *B*–*D*.

**Figure 3.**
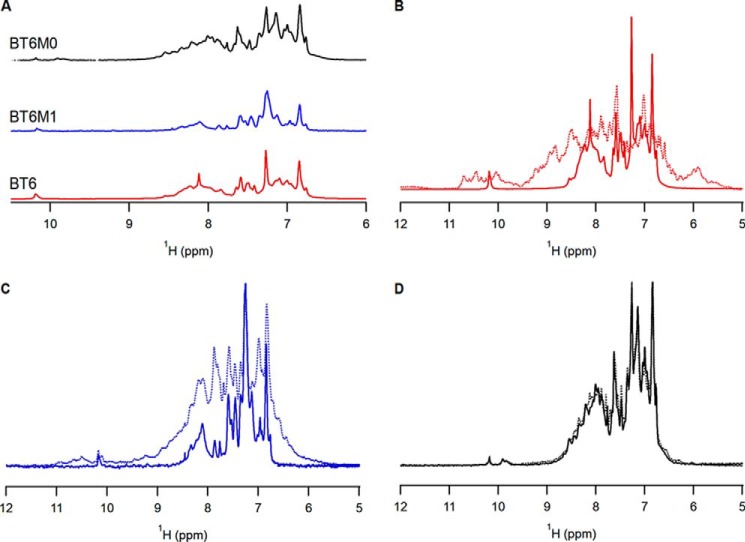
**Proton NMR resonances of maquettes with and without heme cofactor.** *A–D*, the amide proton region for apo-BT6 (*red*), apo-BT6M1 (*blue*), and apo-BT6M0 (*black*) (*A*), and comparisons in the absence (*solid line*) and presence (*dashed line*) of heme for BT6 (*B*), BT6M1 (*C*), and BT6M0 (*D*). For BT6 and BT6M1, the presence of heme induces changes in the amide proton resonance dispersion, whereas for BT6M0 negligible changes are observed. These chemical shift changes are consistent with a heme binding event coupled with a change in protein conformation.

One-dimensional proton (^1^H) NMR spectroscopy was used to assess conformational changes in tertiary structure upon heme binding to maquettes. A ^1^H NMR spectrum characteristic of a protein with limited tertiary structure was observed for all maquette scaffolds in the absence of heme (Fig. 3*A*, Fig. S3). Addition of heme to BT6 showed greater dispersion of resonances in the amide proton region, with a notable increase in the number of peaks at around 10 ppm, suggesting that the heme is binding to the scaffold and stabilizing the protein tertiary structure (Fig. 3*B*). BT6M1 showed a small increase in resonance dispersion following heme addition (Fig. 3*C*), whereas BT6M0 showed no significant change in amide proton dispersion (Fig. 3*D*). The increase in the chemical shift dispersion observed in the methyl proton region (at around 1.0–0.0 ppm) for BT6, BT6M1, and BT6M0 mirrored the behavior observed for the amide proton resonances (Fig. S4). Together, the increase in ^1^H NMR dispersion in both backbone amide and methyl regions confirm the ligation of heme into the BT6 and BT6M1 variants and indicate an increase in protein folding upon ligation. These results are consistent with a previous NMR study that showed poor dispersion for a related apo-maquette, and progressive structuring of the maquette when 1 then 2 eq of heme were added (28).

### In vivo Tat export assays

The *E. coli* TorA signal peptide is sufficient to direct green fluorescent protein (GFP) to the *E. coli* periplasm via the Tat system (13). Constructs in which the sequence encoding the TorA signal peptide (residues 1–39) and first 4 amino acids of the mature TorA protein (residues 40–43) were added in-frame to the 5′ terminus of the genes encoding the BT6 maquette variants followed by a C-terminal His_6_ tag (Fig. S5) were synthesized and cloned into the pEXT22 vector (Table S1). In the TorA–BT6 variants residues His-95 and His-130 correspond to His-53 and His-88 in the maquette scaffolds presented in Fig. 1.

The resulting plasmids were co-transformed into *E. coli* BL21(DE3) along with empty pET-21a(+). The pEXT22 vector was used as it allows isopropyl β-d-1-thiogalactopyranoside (IPTG)-inducible expression of the synthetic gene under the control of a tightly regulated *tac* promoter. The low copy number of pEXT22 (R100 origin of replication, 1–1.5 copies per chromosome) (32, 33) limits production of recombinant protein, to avoid overwhelming the *E. coli* Tat apparatus with substrate.

**Figure 4.**
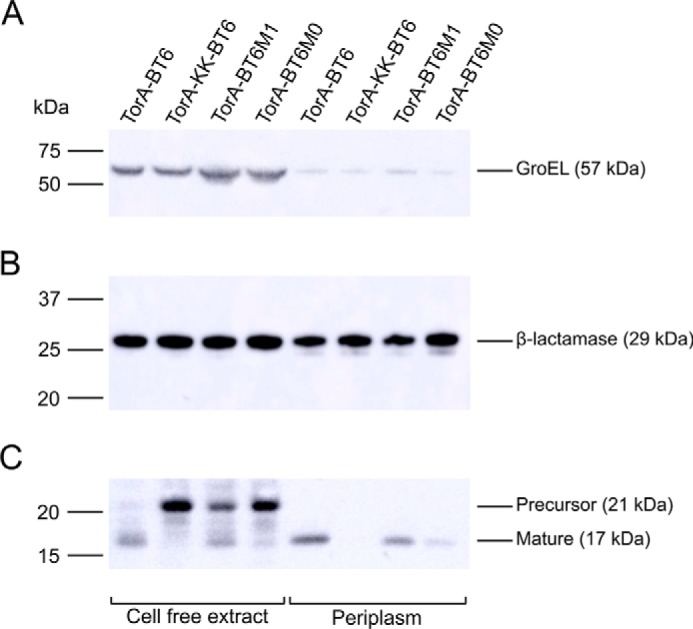
**Differential export of BT6 variants by the Tat system determined by immunoblotting.** *A,* GroEL was used as a cytoplasmic marker to confirm only very minor cytoplasmic contamination of periplasmic preparations. *B,* β-lactamase was used to confirm equal loadings of cell-free extracts or periplasm samples. *C,* the C-terminal His tag on the BT6 maquettes was used to determine the degree of Tat-dependent periplasmic localization and the level of the unprocessed precursor proteins in cell-free extracts. For all panels the positions of molecular weight markers and the expected size of proteins are indicated alongside the blots. Each blot is representative of at least 3 independent experiments.

Following growth and protein production, periplasmic fractions were prepared from harvested cells as described under “Experimental procedures” and analyzed alongside cell-free extracts prepared from identically grown cells. The C-terminal His_6_ tag allows immunodetection of the recombinant protein; the predicted molecular mass of the unprocessed precursor protein is ∼21 kDa, whereas the Tat-processed mature protein is ∼17 kDa. Anti-GroEL was used to probe the degree of cytoplasmic contamination in the periplasmic preparations (Fig. 4*A*); for all samples only a faint signal was detectable in the periplasm compared with that detected in cell-free extracts, indicating the level of contamination was very low. The Tat export assays were performed in cells that also maintained the pET-21a(+) vector, allowing immunoblot signals to be normalized by immunodetection of β-lactamase (Fig. 4*B*), allowing direct comparison of the degree of Tat transport between samples.

For TorA–BT6, a 17-kDa signal was present for both periplasmic and cell-free extract (Fig. 4*C*) preparations with no signal detected at 21 kDa, indicating complete processing and export of TorA–BT6. To confirm the periplasmic localization was Tat-dependent, a R12K/R13K (KK) variant of the TorA signal peptide was generated and fused to BT6 (Table 1); it has previously been demonstrated that the KK motif completely abolishes Tat-transport (12, 34). When this construct was tested, a 21-kDa signal corresponding to the unprocessed apoprotein is observed in blots of total lysates and no processed protein was detectable in the periplasm (Fig. 4*C*). Together these data indicate that the BT6 scaffold is able to acquire heme from the native biosynthetic pathway and fold *in vivo*, and that this folding event can be detected by the Tat system, with the TorA signal peptide sufficient for Tat-mediated recognition and transport of heme-loaded BT6 to the *E. coli* periplasm.

To probe the proofreading ability of the Tat machinery to recognize and efficiently export only folded proteins, similar constructs were generated in which the TorA signal peptide was fused to the BT6M1 and BT6M0 maquettes, which, in the presence of heme, are either partially folded (BT6M1) or unfolded (BT6M0) relative to BT6 (Figs. 2 and 3). Compared with BT6, considerably less protein was found in the periplasm for BT6M1, and almost none for BT6M0 (Fig. 4*C*). Conversely, blots of cell-free extracts of the same strains reveal a significant signal for the unprocessed maquettes for BT6M0, and to a lesser extent BT6M1, as observed for the TorA–KK–BT6 control, showing that the preprotein accumulates in the cytoplasm when it is less well folded (Fig. 4*B*).

The cellular levels of maquette variants should be very similar as all the proteins are identical apart from 1 or 2 point mutations and were expressed from the same plasmid in the same cell line under the same conditions. Indeed, the data presented in Fig. 4*C* shows that the total signal (preprotein plus exported protein where relevant) for all three maquettes is similar. However, to further confirm that the differential periplasmic targeting of the three BT6 variants by the Tat system was due to differences in their heme loading and associated folding and not in expression, synthesis, or stability, we performed two additional control experiments. First we produced each maquette under the same conditions as for the Tat export assays but without the TorA signal peptide, and immunoblotting confirmed approximately equal levels of each (Fig. S6*A*), ruling out that BT6M1 and BT6M0 are produced at lower levels or are more rapidly degraded in the cytoplasm than BT6.

We also performed Sec system-mediated export assays of BT6, BT6M1, and BT6M0. As the Sec system transports unfolded proteins across the cytoplasmic membrane, differences in folding, mediated in the case of BT6 by heme binding, will not affect transport. Maquette genes were cloned into the pET-22b(+) vector (Table S1) in-frame with an N-terminal PelB (*Erwinia carotovora* pectate lyase B) Sec leader peptide (22 amino acids) (35, 36) (Table 1). Stop codons were omitted so proteins had a C-terminal His_6_ tag for immunodetection; the unprocessed proteins have a predicted molecular mass of ∼19 kDa and the mature maquettes would be ∼17 kDa following cleavage of the PelB signal sequence. Fig. S6*B* shows that normalized loadings of cell lysates yield single, similarly intense signals for BT6, BT6M1, and BT6M0, indicating all three apo-maquettes are equally synthesized and transported to the periplasm when directed through the Sec system.

### Purification of maquettes from the periplasm

**Figure 5.**
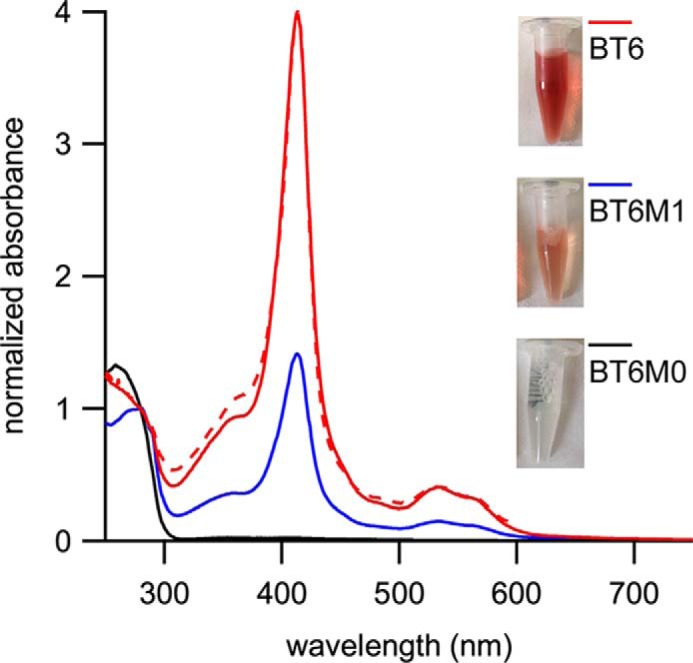
**Spectroscopic analysis of purified maquette proteins.** UV-visible absorption spectra of maquettes purified from the periplasm of *E. coli* (BT6, *red line*; BT6M1, *blue line*; BT6M0, *black line*) compared with BT6 reconstituted with heme *in vitro* (*red dashed line*). Spectra are normalized to absorbance at 280 nm. *Inset panels* show the purified proteins. See Fig. S7 for SDS-PAGE analysis of the purified maquettes.

To demonstrate that heme ligation into BT6 was responsible for its Tat-dependent localization in the periplasm, production of His-tagged maquettes was performed on a larger scale using the recently described *E. coli* W3110-TatExpress cell line (37). Cells were fractionated and maquette proteins were purified from the periplasmic fraction by immobilized metal affinity chromatography. Eluates were concentrated and striking differences in pigmentation between the maquette variants were observed, with a deep red color for BT6, weaker pigmentation for BT6M1, and no visible color for BT6M0 (Fig. 5, *inset panels*). SDS-PAGE of the concentrated eluates revealed the same pattern as the Tat exports presented in Fig. 4, with more BT6 than BT6M1 and only a trace amount of BT6M0 (Fig. S7). This also ruled out any leakage of unprocessed protein across the cytoplasmic membrane as the larger precursor species were not purified (Fig. S7). The absorbance spectra of Tat-exported BT6, BT6M1, and BT6M0 samples normalized at 280 nm are shown in Fig. 5, along with *in vitro* heme-reconstituted BT6. There is a close match between the spectra of the Tat-exported heme-loaded BT6 purified from the periplasm and the reconstituted BT6 sample (Fig. 5, *solid red versus dashed red lines*). The high affinity of BT6 for heme (28) ensures full occupancy of the two heme-binding sites, so the Tat-transported BT6 purified from the periplasm is also fully heme bound. These data, alongside the export assays shown in Fig. 4, reveal that the TorA–BT6 maquette is able to sequester heme *b* from the native *E. coli* biosynthetic pathway, correctly fold around its substrate, and retain the pigment following export by the Tat-apparatus.

## Discussion

The Tat pathway is present in the cytoplasmic membranes of most prokaryotic organisms and is evolutionarily conserved in the thylakoids of plant chloroplasts and some mitochondrial membranes (reviewed in Refs. 3 and 7). As well as a biosynthetic role in numerous important cellular processes, the Tat pathway is required for colonization and virulence of globally significant human pathogens (38, 39). The Tat system differs from the universally conserved Sec pathway, which translocates unstructured polypeptides, as it transports folded, typically cofactor-containing proteins. How the Tat apparatus is able to determine that a protein is folded, cofactor loaded, and suitable for export is not understood.

Here, we have used artificial heme maquette proteins to investigate the ability of the archetypal *E. coli* Tat machinery to discriminate between folding states of protein substrates. The maquettes used in this study are four-helix bundle proteins enclosing a hydrophobic cavity that can accommodate heme cofactors ligated by histidine residues. Point mutations to the BT6 maquette, affecting only single histidine ligands, generated the BT6M1 and BT6M0 maquettes, and were sufficient to reduce the number of bound hemes, verified by absorption spectroscopy (Fig. 2*a*). The number of bound hemes therefore provides a simple, well-defined method to alter the stability of a protein substrate. The consequent effects of heme content on the thermostability and folding of each maquette were measured using temperature-dependent CD spectroscopy (Fig. 2*b*, Table 2) and ^1^H NMR (Fig. 3).

When directed for export through the Tat export machinery, there were clear differences in the extent of translocation for BT6, BT6M1, and BT6M0 maquettes (Fig. 4) that correlate with the *in vitro* stability and folding experiments for heme-bound constructs. Export efficiency decreased with increasing conformational flexibility; the unstructured BT6M0 maquette is largely rejected for export, whereas limited export was observed in the intermediate case of the single heme-binding BT6M1 maquette.

The export data shown would only be obtained if the BT6 maquettes were able to ligate heme, *in vivo*, as heme coordination is a pre-requisite for protein stability and folding. Evidence for *in vivo* heme ligation was shown with the purification of heme-containing maquettes from the periplasmic fraction of *E. coli* (Fig. 5), the spectra of which strongly resemble those of heme proteins generated *in vitro* (Fig. 2*A*).

Although the native *E. coli* Tat translocase will never have encountered the artificial protein substrates described here, our data show that it is able to recognize and distinguish between them, even to the extent of processing the intermediate state of BT6M1 differently from the folded BT6 and the least structured BT6M0. Thus, the Tat proofreading process must involve a generic form of discrimination; the correlation between the structural flexibility of the Tat substrate and its suitability for export suggests that there are initial encounters between Tat components and the substrate at the membrane surface. Such interactions might sense flexible motions of the substrate that are transmitted to other components of the Tat machinery, preventing transport across the membrane.

This proof-of-concept study shows that an artificial protein, engineered to bind heme *b* from a native biosynthetic pathway, can be exported from the cell in its correctly folded state. Development of this concept may contribute a significant advance in biotechnology, where the principle could be applied to other organisms and to important biomolecules and protein-cofactor complexes, particularly those that may cause toxicity to the host.

## Experimental procedures

### Computational prediction and molecular dynamics refinement of protein structures

To generate illustrative structural models for BT6, the amino acid sequence was run through the PEP-FOLD structure prediction algorithm (40) for a total of 100 simulations, from which the five best-scoring structures were selected for further analysis. All five structures were four-helix bundles in agreement with the schematic structure described by Farid *et al.* (28). From these, a structure with both pairs of histidines positioned closest to their respective heme-ligating positions was selected as a starting structure. From this structure, mutants BT6M1 and BT6M0 were generated using PyMOL version 1.7 (PyMOL Molecular Graphics System Version 1.7, Schrödinger, LLC). Hemes (0, 1, or 2) were manually docked into the bis-His sites of the structures.

To relax the starting structure into an energy-minimized conformation, molecular dynamics (MD) pre-processing, production runs, and post-processing were performed with Gromacs version 4.6 (41). MD simulations were performed using the CHARMM27 force field for proteins and TIP3P-CHARMM model for water. For the heme cofactor, parameters were used for reduced, deprotonated, bis-His–ligated heme included in the CHARMM27 force field. Covalent bonds were explicitly specified between the heme iron and the ϵ-nitrogen of the relevant histidine residues. NaCl was added to a total concentration of 150 mm. Each structure was equilibrated for 100 ps in the NVT ensemble, followed by 100 ps in the NPT ensemble. Temperature and pressure/density plots (following NVT and NPT equilibrations, respectively) were checked for convergence before proceeding to production MD runs of 50 ns. Following production runs, trajectories were corrected for periodicity and centered on the protein. Snapshots of the trajectories at 50 ns were exported to .pdb files. Electrostatics and solvent-accessible surface calculations were performed using APBS (42). Visualization of calculations and structures was performed with the PyMOL Molecular Graphics System version 1.7.

### DNA manipulation

Plasmids and primers used in this study are provided in Tables S1 and S2, respectively. Synthetic genes encoding maquette protein variants codon optimized for expression in *E. coli* were purchased from DNA2.0 (now ATUM) or Integrated DNA Technologies. Point mutations were generated using the QuikChange II Site-directed Mutagenesis Kit (Agilent). The TorA Tat signal peptide was amplified from *E. coli* genomic DNA and joined in-frame to maquette constructs by overlap extension-PCR using Q5 High-Fidelity DNA Polymerase (New England Biolabs). All plasmids were sequence verified by automated DNA sequencing (GATC Biotech). Competent *E. coli* JM109 (Promega) was used for cloning and was grown in Luria-Bertani (LB) broth/agar supplemented with the appropriate antibiotic(s) (Table S1).

### Production and purification of untagged maquettes

The high copy number pJexpress414 plasmid was used for recombinant protein production under control of an IPTG-inducible T7 promoter. *E. coli* BL21(DE3) containing the desired plasmid was grown with shaking (230 rpm) at 37 °C in LB broth with 100 μg ml^−1^ ampicillin to an absorbance at 600 nm (*A*_600_) of ∼0.6. At this point IPTG was added to a final concentration of 1 mm to induce expression and the cultures were incubated for a further 16 h at 37 °C. Cells were harvested by centrifugation (4,400 × *g*, 15 min, 4 °C) and resuspended in buffer A (50 mm HEPES, pH 7.4, 500 mm NaCl, 5 mm imidazole). Cells were lysed by sonication on ice and the lysate was clarified by centrifugation (53,000 × *g*, 30 min, 4 °C). The supernatant was filtered through a 0.45-μm filter and applied to a Chelating Sepharose Fast Flow column (GE Healthcare) pre-equilibrated with 10 mg ml^−1^ of nickel sulfate. The column was washed with 5 column volumes of buffer A with the flow-through and wash was collected and pooled. The pooled sample was buffer exchanged into buffer B (50 mm HEPES, pH 7.4) and further purified by ion exchange chromatography on a Fast Flow Q-Sepharose column (GE Healthcare) with a linear gradient of 0–1 m NaCl in buffer B. Where required maquettes were further purified by size exclusion chromatography on a Superdex 200 Increase column (GE Healthcare) in buffer C (50 mm HEPES, pH 7.4, 200 mm NaCl). Where necessary protein was concentrated using Vivaspin centrifugal concentrators (Sartorius).

### Heme reconstitution into apo-maquettes

Hemin (Sigma) stocks (1 mg ml^−1^) were prepared in 100% DMSO. Protein concentrations were calculated by absorbance at 280 nm following the method described by Gill and von Hippell (43), and using the experimentally determined extinction coefficient of 32.6 mm^−1^ cm^−1^. Reconstitutions were conducted with a 10-fold molar excess of hemin in buffer D (50 mm HEPES, pH 7.4, 200 mm NaCl, 20% (v/v) DMSO) and incubated for 45 min at 25 °C, before being transferred to ice and buffer exchanged into buffer B using Vivaspin centrifugal concentrators. Unbound cofactor was removed by ion exchange chromatography on a DEAE-Sepharose (Sigma) column.

### UV-visible absorption spectroscopy

Protein samples were buffer exchanged into buffer E (5 mm sodium phosphate buffer, pH 7.4) and UV-visible absorption was measured in a 1-cm path length UV cuvette in a Cary 60 UV-visible spectrophotometer (Agilent) at room temperature.

### CD spectroscopy

Mean residue ellipticity ([θ]_MRW_) of protein samples was measured in a 1-mm path length quartz cuvette on a Jasco J-810 spectropolarimeter with a Jasco PFD-425S Peltier to enable temperature control. Spectra were obtained from 15 to 80 °C at 5 °C intervals. Spectra were recorded continuously at a scan speed of 100 nm min^−1^, with 1-nm resolution and a 4-s response with 4 accumulations. Ellipticity ([θ]) at 222 nm was measured every 1 °C from 15 to 80 °C at 1 °C min^−1^ with a 4-s response. Melting temperatures (*T_m_*) and refolding temperatures (*T_f_*) are the temperature at which 50% of the protein is unfolded or folded, respectively, as determined by fitting melting data to a sigmoidal Boltzmann distribution according to the following equation, where *T* is the temperature, *T_m_* is the melting (or folding) temperature, and *T*_0_ is the initial temperature of the experiment.

(1) $${\left[\theta \right]}_{222\text{nm}}={\left[\theta \right]}_{222\text{nm}}^{\text{base}}+\left(\frac{{\left[\theta \right]}_{222\text{nm}}^{\max}}{1+\exp\left(\frac{\frac{T_{m}-T_{0}}{\delta T}}{\delta \left[\theta \right]}\right)}\right)$$

### Proton NMR spectroscopy

Spectra were recorded at 298 K on 0.2–0.5 mm protein samples in buffer E, with the addition of 10% D_2_O (spectrometer lock), and 1 mm trimethylsilyl propanate (reference standard). ^1^H NMR spectra were recorded using a Bruker Avance 800 MHz spectrometer fitted with a 5-mm QXI room temperature probe, equipped with *z* axis gradients. One-dimensional experiments were acquired as accumulations of 4096 transients over a spectral width of 24.038 kHz, corresponding to a proton spectral width of 30.0 ppm. All data were processed using an EM window function and 5-Hz line broadening, without linear prediction in TopSpin (Bruker). Spectra were referenced to trimethylsilyl propanate at 0 ppm prior to overlay and analysis.

### E. coli fractionation

To avoid overloading the Tat system for *in vivo* transport assays, maquette genes were cloned into the KpnI and XbaI sites of the low copy number pEXT22 plasmid for expression from a tightly controlled *tac* promoter (33). The pEXT22 constructs (Table S1) were co-transformed into *E. coli* BL21(DE3) cells along with empty pET-21a(+). Cultures (50 ml) were grown at 37 °C with shaking (230 rpm) in LB medium with 30 μg ml^−1^ of kanamycin and 100 μg ml^−1^ of ampicillin in 250-ml Erlenmeyer flasks. At an *A*_600_ of ∼0.6 expression was induced with 0.5 mm IPTG for 2 h. Cells were harvested by centrifugation (3,900 × *g*, 30 min, 4 °C) and washed with buffer F (100 mm Tris acetate, pH 8.2, 500 mm sucrose, 5 mm EDTA). To prepare cell-free lysates, cells were re-suspended to *A*_600_ ∼2 in 2 ml of chilled Buffer C and lysed by sonication on ice. The supernatant, following clarification by centrifugation (16,600 × *g*, 30 min 4 °C), was collected as the cell-free extract. Periplasmic fractions were obtained using a procedure based on the EDTA/lysozyme/osmotic shock method described by Randall and Hardy (44). Briefly, cells were re-suspended to *A*_600_ ∼10 in 500 μl of chilled Buffer F followed by the addition of 5 μl of 1 mg ml^−1^ lysozyme. 500 μl of chilled QH_2_O was added and cells were incubated on ice for 5 min prior to the addition of 20 μl of 1 m MgSO_4_. Spheroplasts were pelleted by centrifugation at 16,600 × *g* for 30 min in a pre-chilled (4 °C) microcentrifuge and the supernatant was collected as the periplasm.

### Immunoblotting

Immunoblotting was performed essentially as described previously (45). Briefly, proteins were separated by SDS-PAGE on 12% BisTris gels (Invitrogen), transferred to polyvinylidene difluoride membranes (Invitrogen), and incubated with anti-His_6_ (Bethyl Laboratories, Inc.), anti-β-lactamase (Abcam), or anti-GroEL (Sigma) primary antibodies followed by an appropriate horseradish peroxidase-conjugated secondary antibody (Sigma). Chemiluminescence was detected using the WESTAR ETA C 2.0 chemiluminescent substrate (Cyanagen) on an Amersham Biosciences Imager 600 (GE Healthcare).

### Purification of Tat-exported proteins from E. coli periplasm

The *E. coli* W3110 TatExpress cell line (37) was transformed with the pEXT22 vector containing TorA–BT6, TorA–BT6M1, or TorA–BT6M0 (Table S1). 500-ml cultures were grown in 2-liter Erlenmeyer flasks at 30 °C with 220 rpm agitation. At an *A*_600_ of ∼0.6, protein production was induced with 0.5 mm IPTG and cultures were incubated for 24 h, after which cells were harvested by centrifugation (3,900 × *g*, 30 min, 4 °C). To obtain periplasmic fractions, cells were resuspended in 10 ml of chilled buffer F. 10 ml of chilled milliQH_2_O was added followed by 800 μl of 1 mg ml^−1^ of lysozyme and samples were incubated on ice for 10 min. 800 μl of 1 m MgSO_4_ was added and the solution was centrifuged (16,600 × *g*, 30 min, 4 °C) with the supernatant collected as the periplasm.

Periplasmic fractions were applied to a Chelating Sepharose Fast Flow column (GE Healthcare) pre-equilibrated with 10 mg ml^−1^ of nickel sulfate. The column was washed with 20 ml of buffer A and 20 ml of buffer G (50 mm HEPES, pH 7.4, 500 mm NaCl, 50 mm imidazole). Protein was eluted with 10 ml of buffer H (50 mm HEPES, pH 7.4, 100 mm NaCl, 400 mm imidazole) and the elution fractions were collected.

## Author contributions

G. A. S., K. J. G., N. B. P. A., D. M. J. M., A. S. J., A. J. R., D. B. A., A. A. B., F. S., P. T., P. D., P. L. D., C. R., A. H., and C. N. H. data curation; G. A. S., K. J. G., N. B. P. A., D. M. J. M., A. S. J., A. J. R, D. B. A., A. A. B., F. S., P. T., P. D., P. L. D., C. R., A. H., and C. N. H. formal analysis; G. A. S., K. J. G., N. B. P. A., D. M. J. M., A. S. J., A. J. R, D. B. A., A. A. B., F. S., P. T., P. D., P. L. D., C. R., A. H., and C. N. H. methodology; G. A. S., K. J. G., A. H., and C. N. H. writing-original draft; G. A. S., N. B. P. A., A. H., and C. N. H. writing-review and editing; K. J. G., A. S. J., F. S., P. T., P. D., P. L. D., C. R., A. H., and C. N. H. investigation; N. B. P. A., F. S., P. T., and P. D. software; K. J. G., A. S. J., F. S., P. T., P. D., P. L. D., C. R., A. H., and C. N. H. conceptualization; C. N. H. project administration.

## Supplementary Material

Supporting Information

## Footnotes

9 The abbreviations used are:

Sec: general secretion
Tat: twin-arginine translocase
IPTG: isopropyl β-d-1-thiogalactopyranoside
BisTris: 2-[bis(2-hydroxyethyl)amino]-2-(hydroxymethyl)propane-1,3-diol (systematic).
