# Supplementary material for: Probing the quality control mechanism of the Escherichia coli twin-arginine translocase with folding variants of a de novo–designed heme protein
Source: J Biol Chem. 2018 Mar 20;293(18):6672–81. doi: 10.1074/jbc.RA117.000880 (PMC5936819; doi:10.1074/jbc.RA117.000880)
Supplement: Supporting Information [file supp_293_18_6672__index.html]

Probing the quality control mechanism of the Escherichia coli twin-arginine translocase with folding variants of a de novo-designed heme protein — Protein fold recognition by the Twin Arginine Translocase — Probing the quality control mechanism of the Escherichia coli twin-arginine translocase with folding variants of a de novo–designed heme protein — Protein-fold recognition by the twin-arginine translocase — Supporting Information 

# Probing the quality control mechanism of the *Escherichia coli* twin-arginine translocase with folding variants of a *de novo*–designed heme protein

## Supporting Information

- Supporting information - Supplementary information to be published online
