# Supplementary material for: Probing the quality control mechanism of the Escherichia coli twin-arginine translocase with folding variants of a de novo–designed heme protein
Source: J Biol Chem. 2018 Mar 20;293(18):6672–81. doi: 10.1074/jbc.RA117.000880 (PMC5936819; doi:10.1074/jbc.RA117.000880)
Supplement: Supporting Information [file supp_RA117.000880_133804_2_supp_96527_p5mzl5.pdf]

Supplementary information (7 figures and 2 tables)

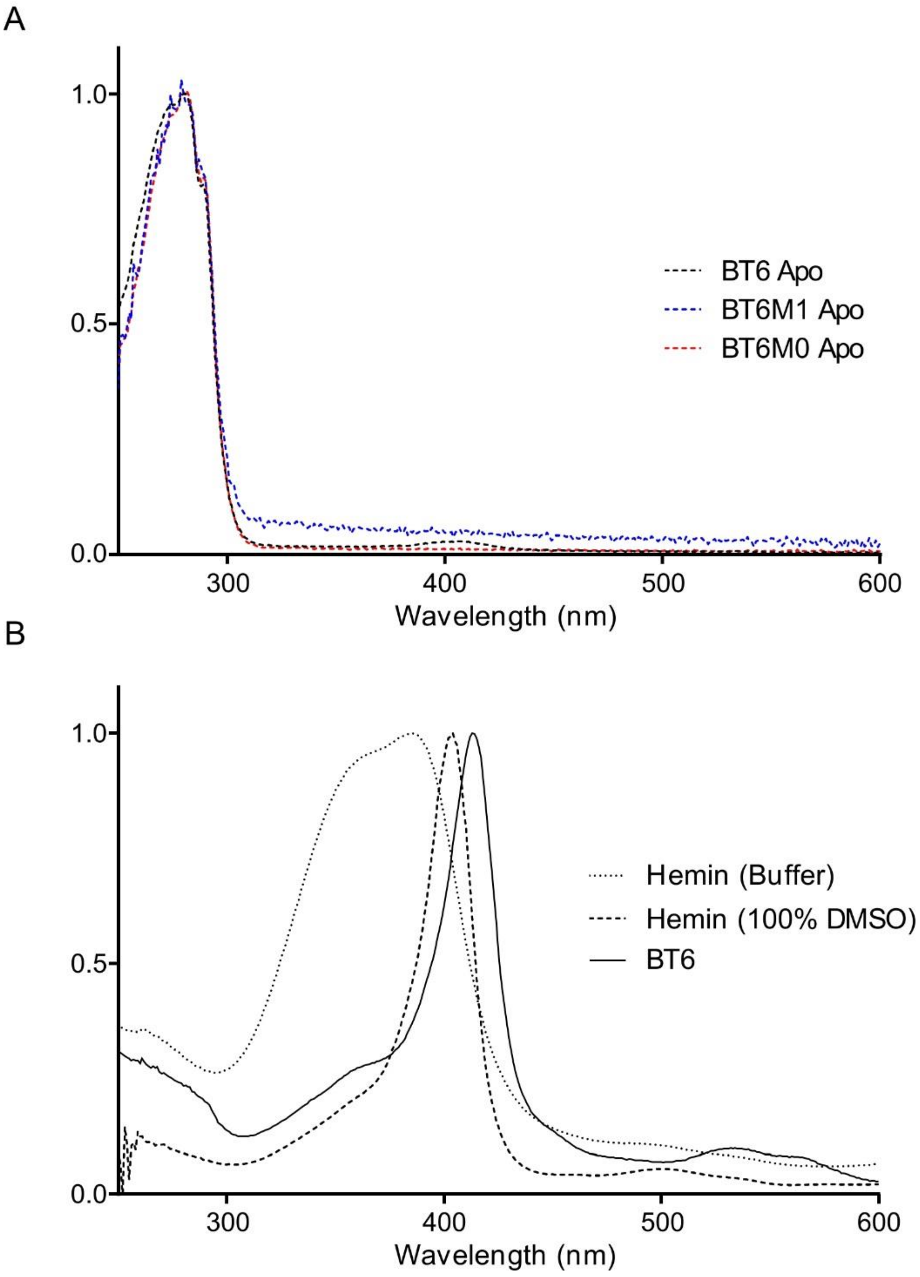

Fig. S1. Absorbance spectra of apo-maquettes and heme in various environments. (A) UV-visible absorbance spectra of purified maquette proteins from the BL21(DE3)-pJexpress overproduction system described in Materials and Methods. **(B)** UV-visible absorbance spectra of hemin in: 100% DMSO (dashed line), aqueous buffer (50 mM HEPES pH 7.4, 200 mM NaCl) (dotted line), and reconstituted in the BT6 maquette (solid line). All spectra normalized to maximum absorption value.

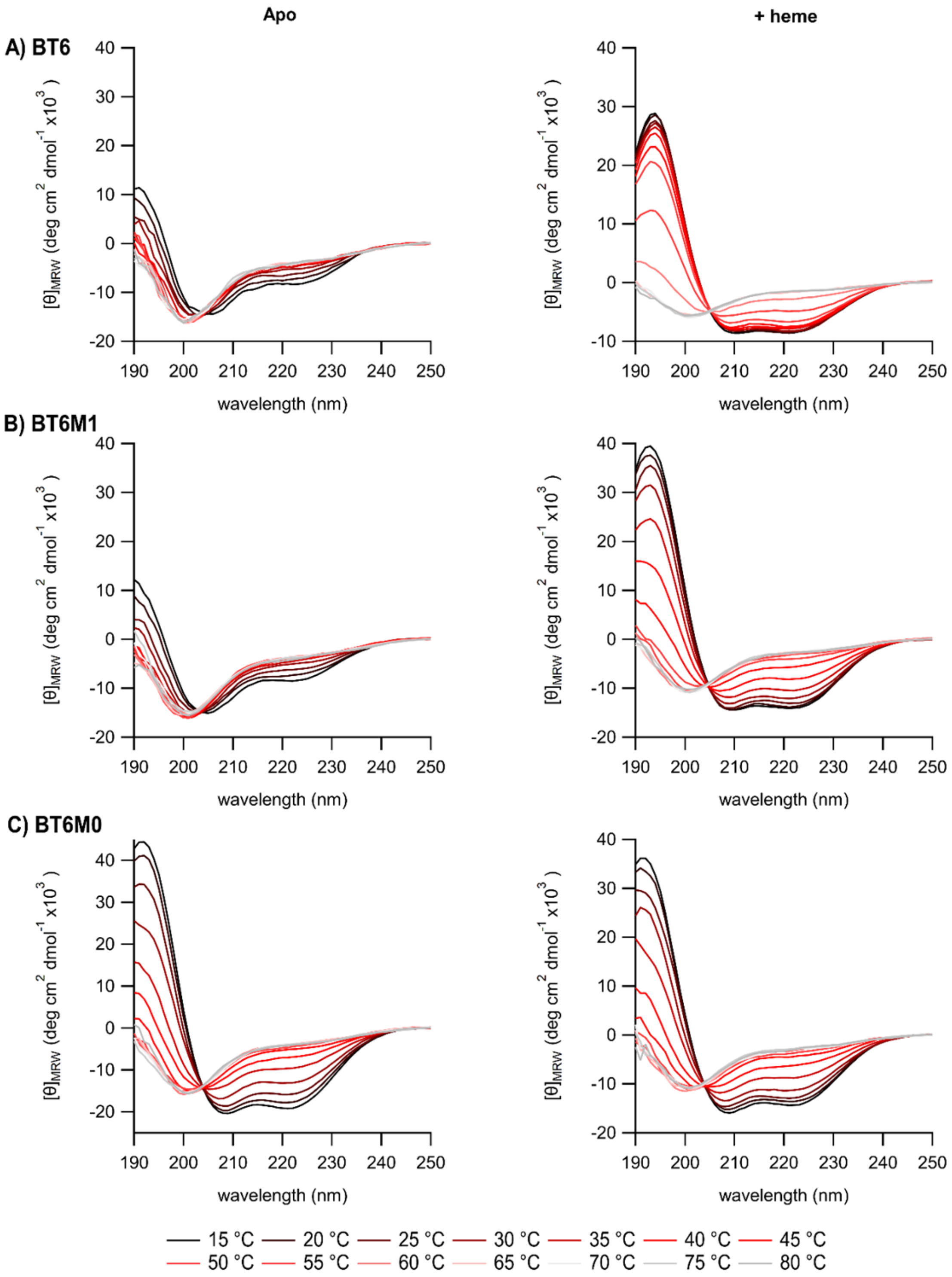

Fig. S2. Circular dichroism mean residue ellipiticity spectra of maquette variants in the absence (left panels) or presence (right panels) of heme for BT6 (A), BT6M1 (B) and BT6M0 (C).

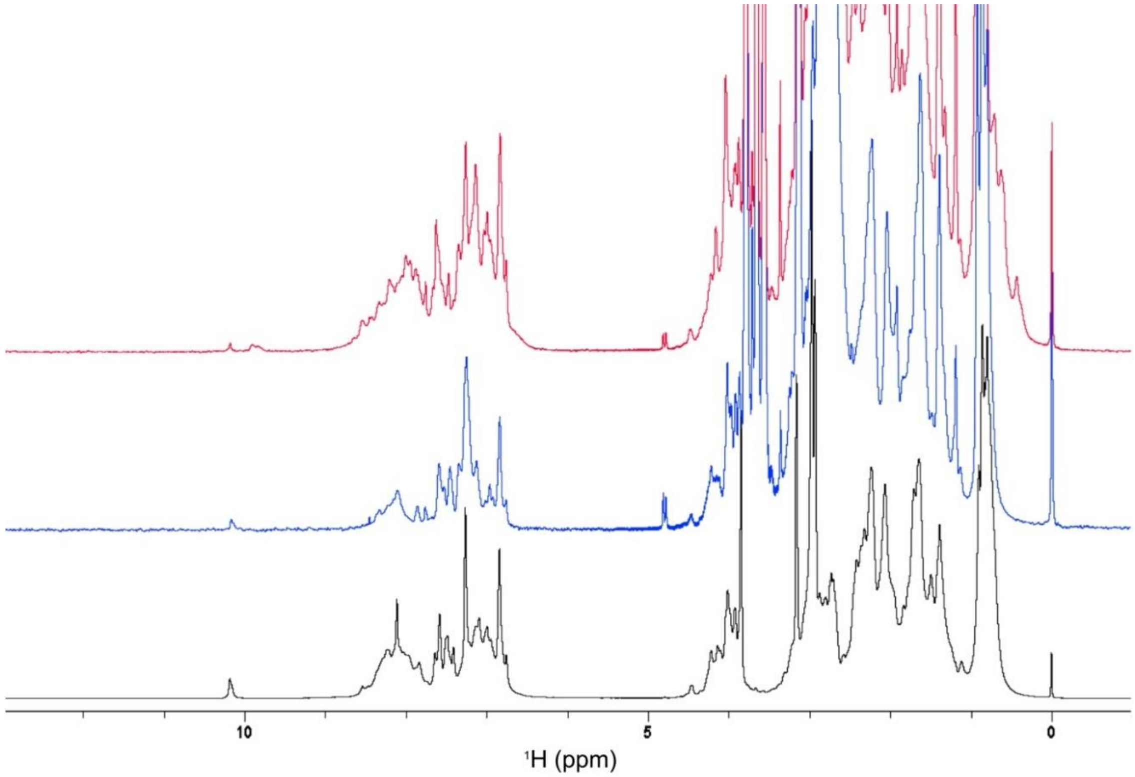

Fig. S3. Full proton NMR resonance spectra of apo-maquette constructs. BT6 (black), BT6M1 (blue) and BT6M0 (red).

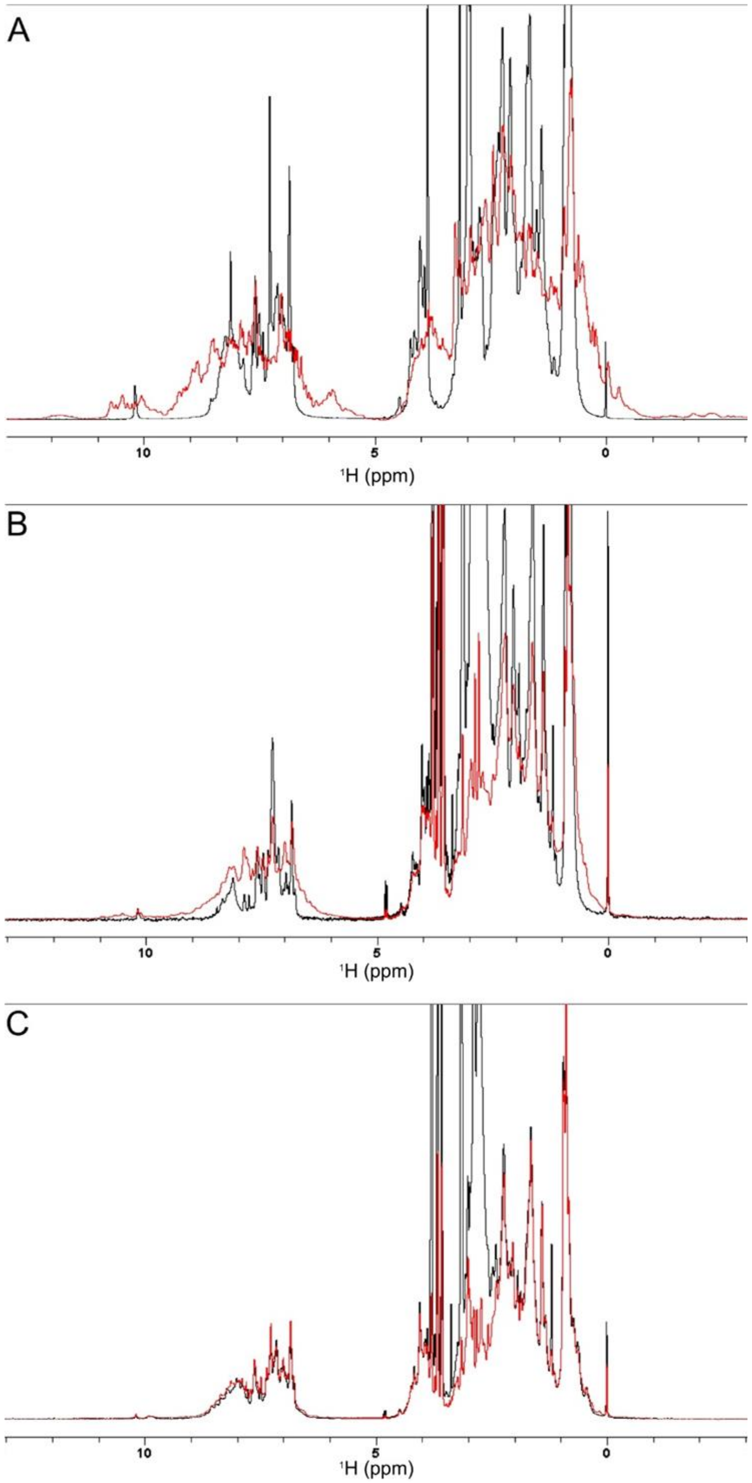

Fig. S4. Full proton NMR resonance spectra for maquette constructs in the absence and presence of heme. (A) BT6, **(B)** BT6M1, **(C)** BT6M0. Apo protein spectra are in black and spectra in the presence of heme in red.

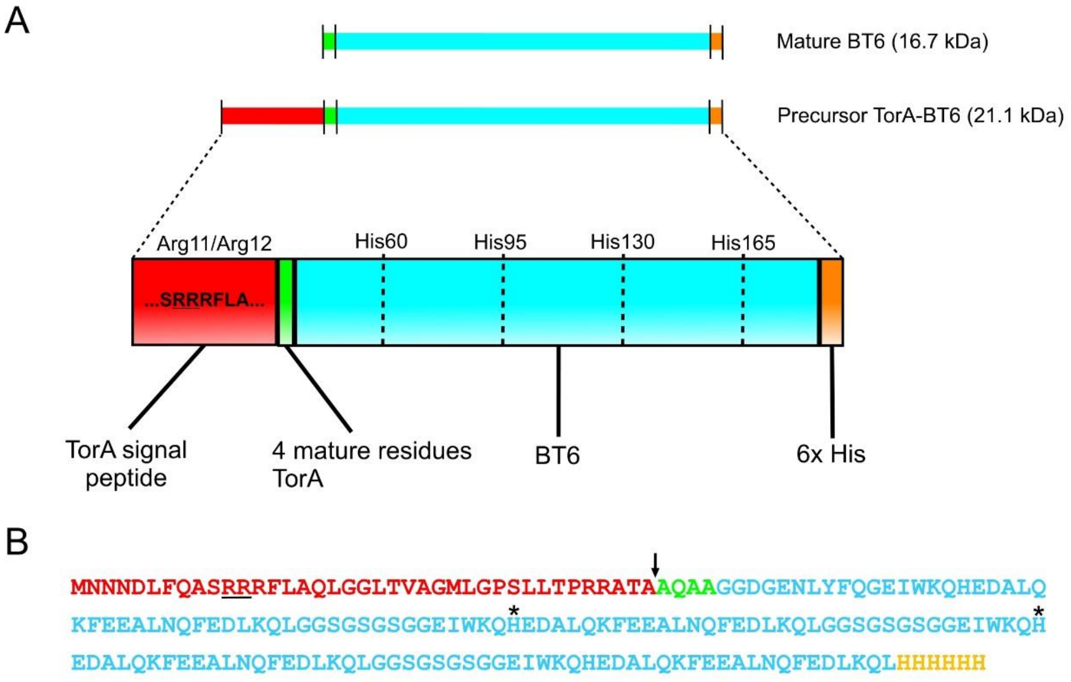

Fig. S5. Schematic representation (A) and amino acid sequence (B) of the TorA-BT6 fusion protein. The 39 amino acid TorA Tat signal peptide is shown in red, the first four mature residues of TorA, included to maintain the signal peptidase cleavage site (indicated by the arrow in panel (B)) in green, BT6 in cyan and the C-terminal His tag in orange. In (A) the predicted molecular weight of the pre and mature proteins is indicated and the positions of the signal peptide twin arginines and heme coordinating histidine residues are indicated. In (B) the twin arginine residues are underlined and the position of the histidine residues that are substituted to alanine in BT6M1 and BT6M0 are shown by asterisks.

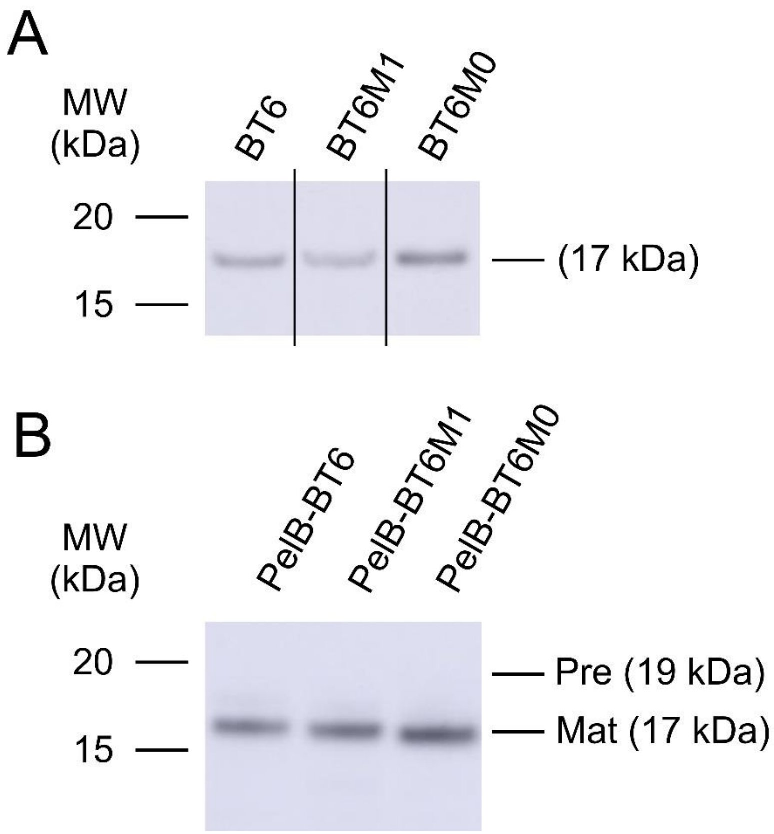

Fig. S6. Immunoblots of maquettes following cytoplasmic production (A) or Sec-translocation (B). Immunodetection of maquettes via the His-tag in cell free extracts of *E. coli* cells producing the indicated maquette lacking a signal peptide (A) or PelB-maquette fusion protein (B). For each panel equal amounts of total protein are loaded for each lane. The positions of molecular weight markers and the predicted size of the precursor (panel B only) and mature proteins are indicated alongside the blots. The vertical dividing lines in panel A indicate where the image has been spliced; all signals were from an identical original image and have not been altered.

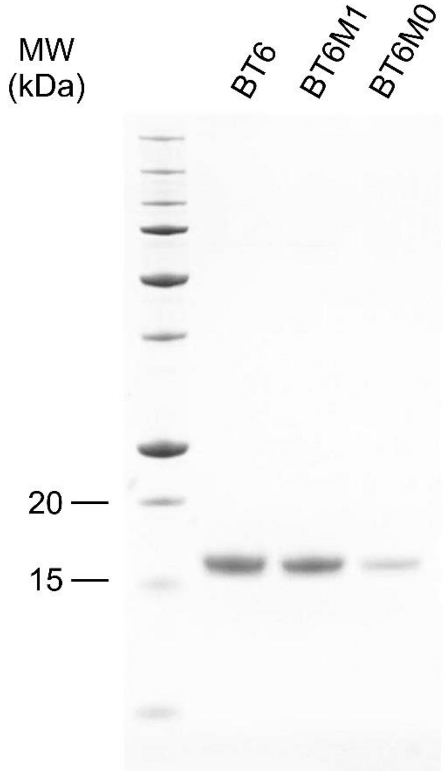

Fig. S7. SDS-PAGE analysis of maquettes purified from *E. coli* periplasmic fractions. Periplasm was prepared from equal amounts of *E. coli* cells and maquettes were purified via their C-terminal His-tag by IMAC. Equal volumes of concentrated eluate were loaded. For spectra and images of the purified proteins see Figure 5.

Table S1. Plasmids used in this study.

**Plasmid**	**Description‡**	**Source/reference**
pJexpress414	High copy number, IPTG inducible T7 promoter, Amp^{R}.	DNA2.0 (now ATUM)
pJexpress414::BT6	BT6 with C-terminal His6 tag cloned into *Nde*I and *Xho*I sites of pJexpress414.	This study
pJexpress414::BT6M1	Quikchange using pJexpress414::BT6 as template to generate BT6M1 variant.	This study
pJexpress414::BT6M0	BT6M0 with C-terminal His6-tag cloned into *Nde*I and *Xho*I sites of pJexpress414.	This study
pJexpress414::BT6_STOP	Quikchange on pJexpress414::BT6 template to introduce stop codon for untagged protein production.	This study
pJexpress414::BT6M1_STOP	Quikchange on pJexpress414::BT6M1 template to introduce stop codon for untagged protein production.	This study
pJexpress414::BT6M0_STOP	Quikchange on pJexpress414::BT6M0 template to introduce stop codon for untagged protein production.	This study
pEXT22	Low copy number (R100 *ori*), IPTG inducible *tac* promoter, *lacI*^{q}, Kan^{R}.	(32)
pEXT22::TorA-BT6	TorA-BT6 with C-terminal His6-tag cloned into *Kpn*I and *Xba*I sites of pEXT22.	This study
pEXT22::TorA-KR-BT6	TorA signal peptide KR version generated by Quikchange using pEXT22::TorA-BT6 as template.	This study
pEXT22::TorA-KK-BT6	TorA signal peptide KK version generated by Quikchange using pEXT22::TorA-KR-BT6 as template.	This study
pEXT22::TorA-BT6M1	TorA-BT6M1 with C-terminal His6-tag cloned into *Kpn*I and *Xba*I sites of pEXT22.	This study
pEXT22::TorA-BT6M0	TorA-BT6M0 with C-terminal His6-tag cloned into *Kpn*I and *Xba*I sites of pEXT22.	This study
pEXT22::BT6	BT6 with C-terminal His6-tag cloned into *Kpn*I and *Xba*I sites of pEXT22.	This study
pEXT22::BT6M1	BT6M1 with C-terminal His6-tag cloned into *Kpn*I and *Xba*I sites of pEXT22.	This study
pEXT22::BT6M0	BT6M0 with C-terminal His6-tag cloned into *Kpn*I and *Xba*I sites of pEXT22.	This study
pET-21a(+)	Optional N-terminal T7-tag and C-terminal His-tag, Amp^{R} (*bla* gene) for immunoblot loading control.	Novagen
pET-22b(+)	N-terminal PelB signal sequence, optional C-terminal His$_{6}$-tag, IPTG inducible T7 promoter. Amp^{R}.	Novagen
pET-22b(+)::BT6	BT6 cloned into *Nco*I and *Xho*I sites of pET-22b(+) so has N-terminal PelB signal peptide and C-terminal His$_{6}$-tag.	This study
pET-22b(+)::BT6M1	BT6M1 cloned into *Nco*I and *Xho*I sites of pET-22b(+) so has N-terminal PelB signal peptide and C-terminal His$_{6}$-tag.	This study
pET-22b(+)::BT6M0	BT6M0 cloned into *Nco*I and *Xho*I sites of pET-22b(+) so has N-terminal PelB signal peptide and C-terminal His$_{6}$-tag.	This study

‡ Amp^{R} = ampicillin resistant; Kan^{R} = kanamycin resistant.

Table S2. Primers used in this study.

Name	Sequence 5'-3'	Details
BT6_F	GGAATTC**CATATG**GGCGGCGACGGCGAGAATC	*Nde*I site in bold.
BT6M0_F	GGAATTC**CATATG**GGCGGCGACGGCGAAAAC	*Nde*I site in bold.
BT6-H53A_S	GAGATCTGGAAGCAGGCCGAGGATGCGCTGCA	H53A Quikchange primers.
BT6-H53A_AS	TGCAGCGCATCCTCGGCCTGCTTCCAGATCTC	
BT6_His_R	CTGG**CTCGAG**TTAATGGTGGTGATGATGGTGCAATTGCTTCAGATCTTCAAATTGG	*Xho*I site in bold; His tag sequence underlined.
BT6M0_His_R	CTGG**CTCGAG**TTAATGGTGGTGATGATGGTGCAGCTGTTTCAGATCCTCAAACTGA	*Xho*I site in bold; His tag sequence underlined.
BT6-STOP_S	TGAGGATCTGAAATAGCTGCACCATCATCACC	Q142STOP Quikchange primers.
BT6-STOP_AS	TGATGATGGTGCAGCTATTTCAGATCC	
torA_F	GGAATTC**CATATG**AACAATAACGATCTCTTTCAGG	*Nde*I site in bold.
torA-BT6_R_OE	*CTCGCCGTCGCCGCC*CGCCGCTTGCGCCGCAGTCGCAC	OLE-PCR primer. Start of BT6 in italics, end of TorA signal peptide underlined.
torA-BT6_F_OE	GCGGCGCAAGCGGCG*GGCGGCGACGGCGAGAATCTG*	OLE-PCR primer. End of TorA signal peptide underlined, start of BT6 in italics.
torA-BT6M0_R_OE	*TTCGCCGTCGCCGCCCG*CCGCTTGCGCCGCAGTCGCAC	OLE-PCR primer. Start of BT6M0 in italics, end of TorA signal peptide underlined.
torA-BT6M0_F_OE	GCGGCGCAAGCGGCG*GGCGGCGACGGCGAAAACC*	OLE-PCR primer. End of TorA signal peptide underlined, start of BT6M0 in italics.
TorA-KR_S	TGTGCCAGAAAACGCCGCTTTGATGCCTGAAAGAGATCGTTATTG	R12K Quikchange primers.
TorA-KR_AS	CAATAACGATCTCTTTCAGGCATCAAAGCGGCGTTTTCTGGCACA	
TorA-KK_S	GATCTCTTTCAGGCATCAAAGAAGCGTTTTCTGGCACAACTC	R13K Quikchange primers.
TorA-KK_AS	GAGTTGTGCCAGAAAACGCTTCTTTGATGCCTGAAAGAGATC	
torA-BT6-pEXT22_F	TAATAA**GGTACC**CATATGAACAATAACGATCTCTTT	*Kpn*I site in bold.
torA-BT6-pEXT22_R	GGCGGC**TCTAGA**CTCGAGTTAATGGTGGTGATGATG	*Xba*I site in bold.
BT6_22b_F	AATGC**CCATGG**GCGGCGACGGCGAGAATCTG	*Nco*I site in bold.
BT6_22b_R	ATGC**CTCGAG**TTGCTTCAGATCTTCAAATTG	*Xho*I site in bold.
BT6M0_22b_F	AATGC**CCATGG**GCGGCGACGGCGAAAACCTC	*Nco*I site in bold.
BT6M0_22b_R	ATGC**CTCGAG**CTGTTTCAGATCCTCAAACTG	*Xho*I site in bold.
